# An emerging paradigms on cervical cancer screening methods and devices for clinical trails

**DOI:** 10.3389/fpubh.2022.1030304

**Published:** 2022-10-28

**Authors:** Kumudha Raimond, Gadudasu Babu Rao, Sujitha Juliet, S. Rubeena Grace Tamilarasi, P. S. Evangelin, Limson Mathew

**Affiliations:** ^1^Department of Computer Science Engineering, Karunya Institute of Technology and Sciences, Coimbatore, Tamil Nadu, India; ^2^Department of Mechanical Engineering, Karunya Institute of Technology and Sciences, Coimbatore, Tamil Nadu, India; ^3^Department of Electronics and Communication Engineering, Karunya Institute of Technology and Sciences, Coimbatore, Tamil Nadu, India

**Keywords:** cervical cancer, HPV screening, VIA, cervical cytology screening test, machine learning, deep learning

## Introduction

Every year around 5,70,000 women are affected with cervical cancer and over 3,11,000 women die from the disease (1). Although there are techniques of screening in various forms and types around the world, most of the knowledge or the technique does not reach the interior parts of the world, like that which are in developing countries. Most of the rural areas either lack good health care support systems or high-level screening equipment, especially when it comes to cancer screening. Most women notice changes in their body only when the symptoms are severe or close to higher rates of malignancies. Fortunately, different studies have come up with various techniques that are cost effective, simple and efficient. In the following sections, major types of screening and the subcategories of testing are described.

Cervical cancer is curable, unlike the majority of malignancies. There are two methods of prevention. First, through immunization, and second, by routine screening that can find HPV infection or aberrant cells before they become malignant (2). Though infections may be cured within 2 years, 10% of the infections may last longer than 2 years. A chronic infection raises the possibility of getting precancerous or, ultimately, aggressive cancer. However, there is a safe and effective vaccine that can stop HPV 16 and HPV 18 infections. Starting at age nine, vaccinations are preferred for young girls. If a high-grade precancerous disease manifests, it must be surgically removed before developing into cervical cancer.

## Literature survey

Van Baars, studied that the primary screening with high-risk human papillomavirus (hrHPV) detection has been advocated to prevent cervical cancer. While given the chance to self-sample for hrHPV testing, women who are not already attending screening (non-responders) are more likely to participate. Dry Evalyn Brush system is as good for self-sampling compared to physician-taken sample for hrHPV detection and is highly acceptable to women. The fact that this study was conducted in a hospital setting is a drawback. Self-samples were always taken prior to practitioner smears, which is another theoretical restriction (3). Parashari et al. found that the Magnivisualizer has an increased identification rate of early malignant tumors from 60 to 95% when compared to unaided visual inspection. It also has allowed for the detection of 58 percent of low-grade dysplasia cases and 83 percent of high-grade dysplasia cases that would not have been detected by simple visual assessment. The Magnivisualizer has a poorer sensitivity for detecting low-grade dysplasias, although this may not be a severe drawback because most low-grade dysplasias tend to regress even in the absence of treatment (4).

Veena Singh et al., showed that, in resource poor environments where colposcopic services are not offered on a local level, a cost efficient, handheld instrument called magnivisualizer is a useful for identifying cervical precancerous and cancerous lesions. In comparison to Visual inspection with acetic acid (VIA), this device demonstrated higher sensitivity (83 vs. 54%) without sacrificing specificity in the detection of severe precancerous lesions of the cervix. Due to the standard of colposcopy has limited specificity, it leads to unnecessary biopsies, therefore it cannot be used as a substitute (5).

Saleh, found that, in comparison to a Pap smear, VIA is an effective screening tool because it is a simple test with a low cost and great sensitivity. It can be therefore used in low-resource locations as an alternative cervical cancer screening method. The sensitivity of Pap smear was 50.1%, specificity was 93.1%, and its negative and positive predictive values were 89.3 and 65.6%, respectively. VIA's sensitivity was 90%, specificity was 37%, and its prediction accuracy was positive. Fifty-two percent and an 81% negative predictive value. Because of the less PPV of VIA, the issue of multiple false positives, discourages the see-and-treat strategy. Although, PPV linked to incidence, the VIA test's capabilities might increase if a see-and-treat approach were used in a high incidence of cervical cancer in a high-risk area (6).

The findings of Emre Ozgu et al. suggest that TruScreen, has 86.1% of sensitivity, and can be used as a cervical cancer screening test that offers quick results without the requirement for a professional. Because it eliminates the need for pathologists and subjectivity in Pap smear interpretation, Cervical cancer screening is possible with TruScreen, particularly in nations with low socioeconomic level. The effectiveness of screening did not significantly enhance when TruScreen and HPV testing were combined (7).

Muszynski et al. performed a study where Colposcopy alone showed 61% of sensitivity and 80% specificity for identifying high-grade lesions. Zedscan and colposcope together exhibited a sensitivity of 93%−100%, and between a range of 91 and 100% negative predictive value (8). Based on the above literatures, there are certain methodologies and techniques with which cervical cancer screening is done. A detailed explanation of the various methods is discussed in methodology. From the literatures it is also observed that each of the techniques has their own advantages and disadvantages.

## Methodology

There are various ways of screening, testing, and diagnosing cervical cancer. The below mentioned are mostly used for cervical cancer and these are as follows:

- Screening using Tissue Scrapping.
- Screening using Visual inspection with acetic acid (VIA).
- Screening using Devices.
- Screening using artificial intelligence (AI) and machine learning (ML) techniques.
- Screening using Mobile technology.

### Screening using tissue scrapping

Cervical screening checks the health of the cervix. It helps to prevent cancer or treat them if any abnormality is found. In this method, a small portion of the cervical tissues are smeared using swab test brushes and are tested in laboratories for traces of HPV infections. There are two major methods through which this screening takes place, one of the methods is the Pap smear test and the other is HPV-DNA test. The Pap smear test is considered as the golden standard for cervical cancer screening (9).

#### Pap smear

Typically, a pelvic exam is performed in addition to the Pap smear (10, 11). In some circumstances, HPV test may be administered to females older than 30 in place of a Pap smear. Based on the type of test, the doctor either places the cell sample obtained from the woman' cervix onto a glass slide (conventional) or place it in a container containing a specific liquid to preserve the sample (liquid-based) (12).

**Figure 1:**
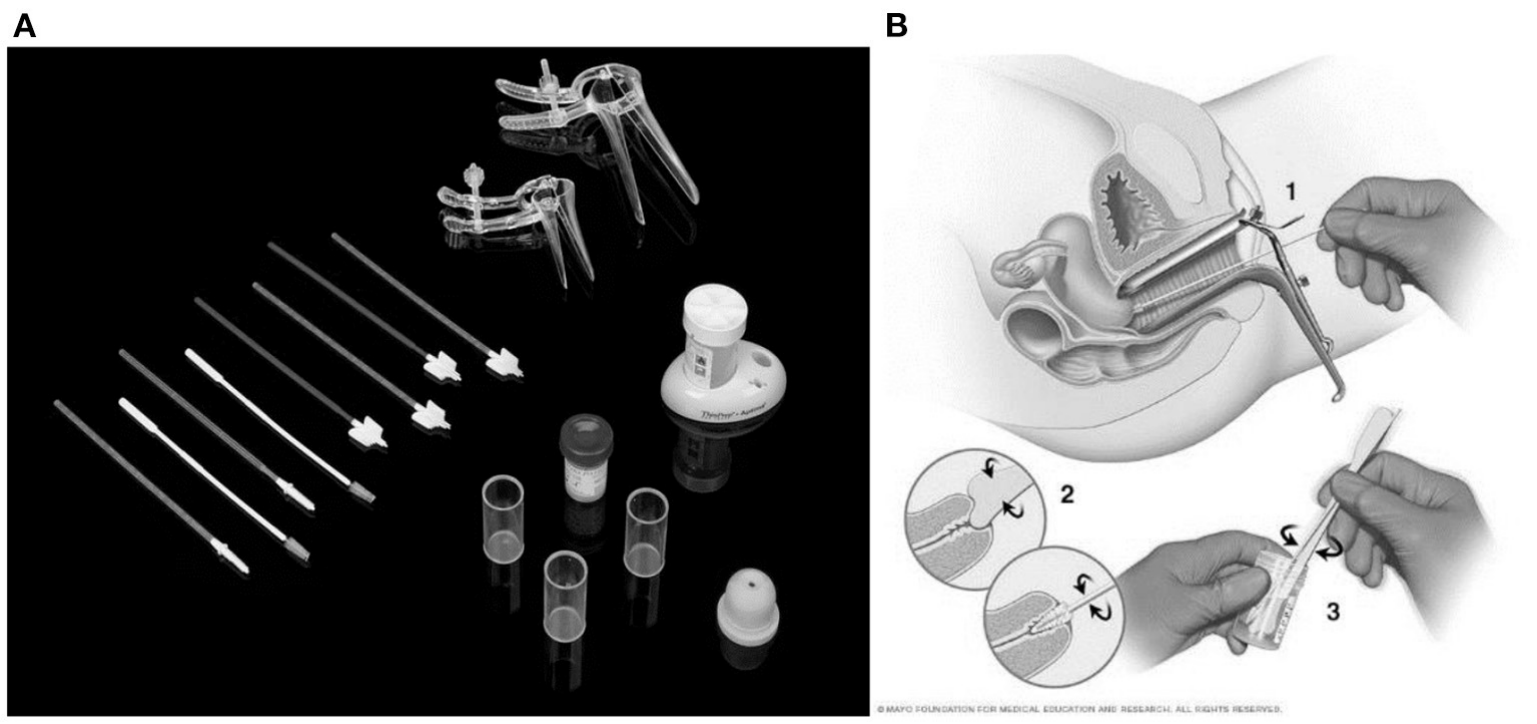
**(A)** The Pap smear test kit. **(B)** The procedure depiction.

Then the samples are then taken to a lab where they are examined under a microscope for cell features that might point to cervical cancer or a precancerous condition. Figure 1A shows the Pap smear test kit and Figure 1B shows the procedure depiction (13).

#### HPV-DNA test

**Figure 2:**
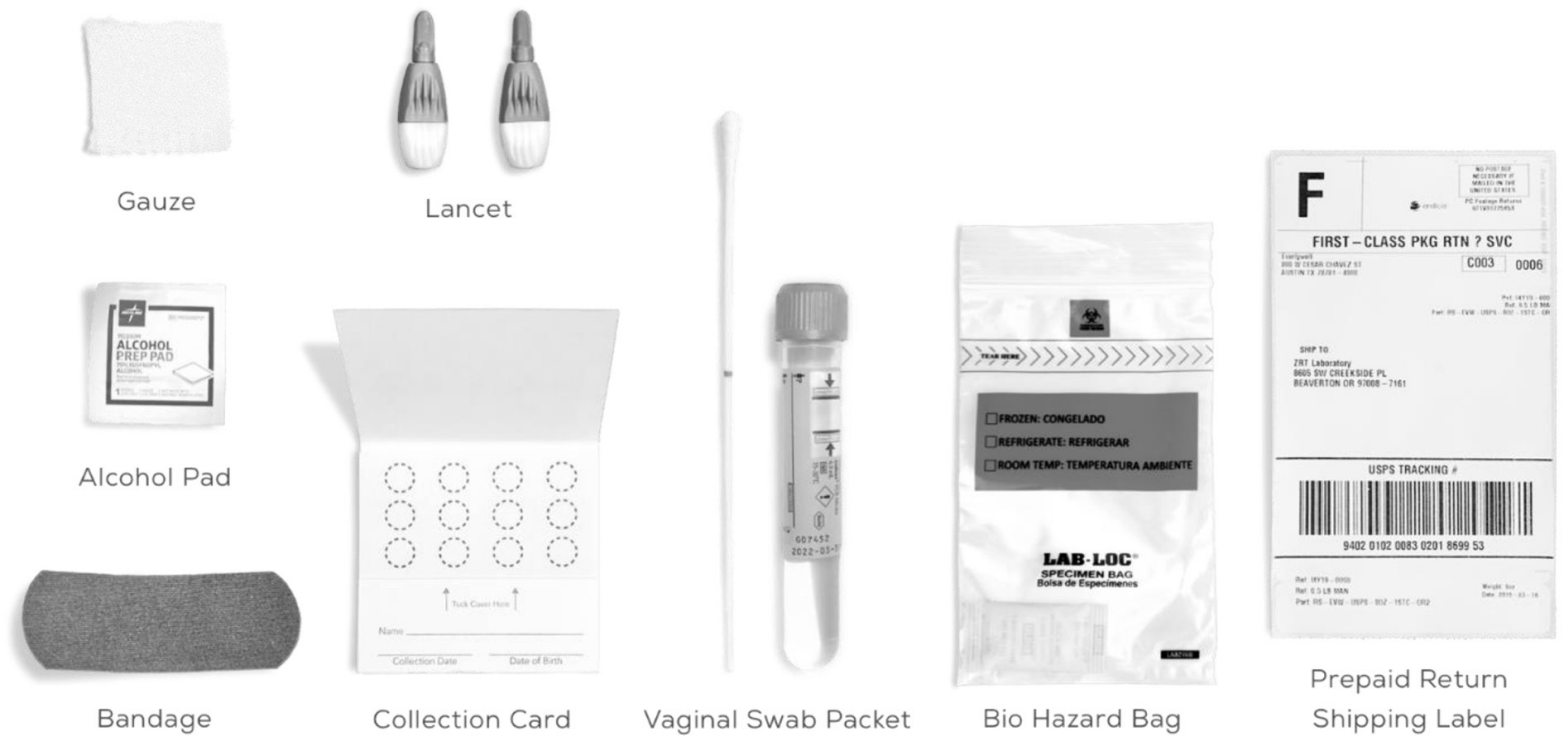
The HPV-DNA test kit.

It is common to have HPV infection around the genitals. Cervical cancer and other malignancies are caused by specific high-risk type of HPV. Low-risk types of HPV may cause genital warts in the vagina, cervix, and on the skin. In general, it is not advised to use the HPV-DNA test to identify low-risk HPV infections. This is because majority of low-risk lesions are physically recognizable. The medical professional inserts a device known as a speculum into the vagina, opens it slightly and gently collects the cells from the area around the cervix (14). Figure 2 shows the HPV-DNA test kit.

The cells are delivered to a lab where a microscope examination will take place. This examiner tests the cells to see if they contain genetic material (referred to as DNA) from cancer-causing HPV strains (15). To identify the exact type of HPV, further testing may be conducted. A Pap smear may be substituted with the HPV DNA test. Co-testing is the term used when they are carried out together.

#### Screening using VIA

**Figure 3:**
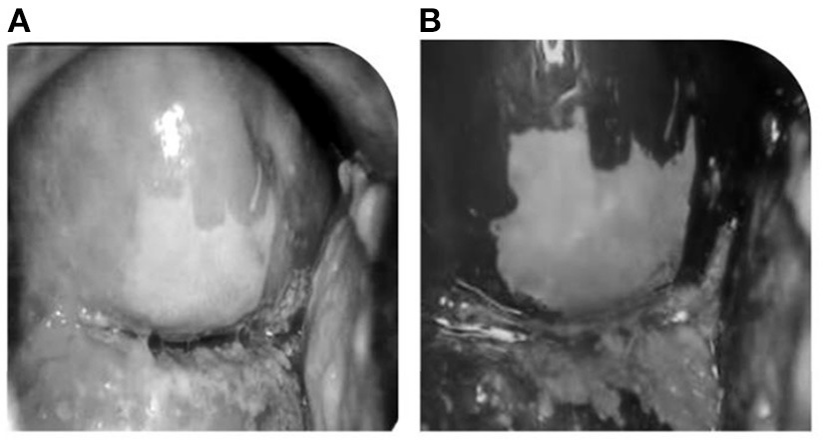
**(A)** Before screening with acetic acid. **(B)** After the acetic screening.

VIA is a screening method in which the cervix is observed after the application of 3%−5% of acetic acid in the cervix region which results in acetowhite lesions. Figures 3A,B shows the result of before and after applying acetic acid. VIA offers the advantages of being simple to use, affordable (16), and sensitive when compared to Pap smear, and quick results assessment (17). As a result, VIA is a good way of cervical cancer screening in many regions of the world, particularly in areas with limited resources. Variations in sensitivity and specificity could be caused by a variety of factors, including the following:

- Expertise training
- Light source variation, and
- The procedure for making a 4%−5% acetic acid solution and storing it.

In poor countries with limited resources, VIA can be utilized as a mass screening method for cervical cancer. It was reported that at the low grade squamous intraepithelial lesion (LSIL) threshold, VIA was less sensitive i.e., is 86.7% which is lesser than that of clinical cytology with a sensitivity of 91.4%, but the difference was not statistically significant (18). HPV testing outperformed cytology in terms of sensitivity, but there was no significant reduction in specificity (84.2 vs. 86.6%).

In addition to VIA tests there are methods that gives importanceto a white light visual inspection of the cervix; white light enables the correct site of biopsy to be selected. The majority of rural clinics utilize a torch or a regular tungsten bulb, which misses many severe lesions. Through this study, the usage of white light is highly advisable for screening purpose. When compared to Pap smear, VIA has a high sensitivity.

### Screening using devices

From the previous studies, it is understood that a clinical test includes the collection of tissues and is slower when compared to other methods. Although the success rates of cancer screening ishigh, the scrapping method may disturb the patient's convenience. In order to be more efficient, cost effective and quick, current studies have come up with techniques that does not involve scrapping of tissues and does not infuse any discomfort. This section will discuss about the modern cervical cancer screening devices and their efficiency.

#### AV Magnivisualizer

It is a low-cost technology for screening uterine cervical cancer using magnivisualizer. It increases the identification rate of early malignant tumors from 60 to 95% when compared to single-handed visual inspection. It also allows the detection of 58% of low-grade dysplasia cases and 83% of high-grade dysplasia cases that would not have been detected by simple visual assessment. The magnivisualizer is highly sensitive, with a sensitivity of around 57.5% in detecting low-grade dysplasia, when compared to 75.3% of cytological evaluation (5). For higher degrees of lesions, however, the two approaches had equivalent sensitivity. The magnivisualizer had a 94.3% specificity, while cytology had a 99% specificity.

**Figure 4:**
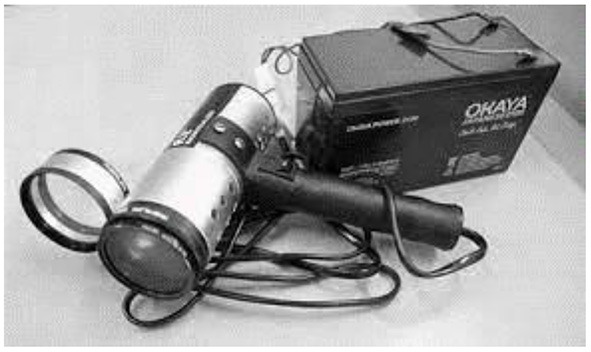
The AV Magnivisualizer.

The AV Magnivisualizer, has a complete spectrum of visible light (white light) and interchangeable magnification. In Figure 4 the AV Magnivisualizer is shown.

The sole accessible light source in primary health Center outdoor situations is usually a tungsten bulb providing yellow light attached to a torch or examination light. On lesions with a pinkish mucosal background, this type of light has a masking effect. The handheld Magnivisualizer can be considered a suitable tool for identification of cervical precancerous and cancerous lesions in low-resource settings where colposcopic services are not available at the community level. However, due to its low specificity, it cannot replace colposcopy, which results in numerous needless biopsies.

#### POCkeT

Point of Care Tampon (POCkeT) is a Novel Low-Cost device that can capture images and can be used to diagnose cervical lesions. By delegating cervical cancer screening to community health workers, the portable, low-cost method has the potential to enhance access to cervical cancer screening in low-resource settings. Women who enter the screening cascade for the first time are usually not familiar with the procedure of having a speculum and are intimidated by the idea of having a cold metal object inside their bodies. This barrier was the reason that ultimately led to the conceptualization of the pocket colposcope (19).

**Figure 5:**
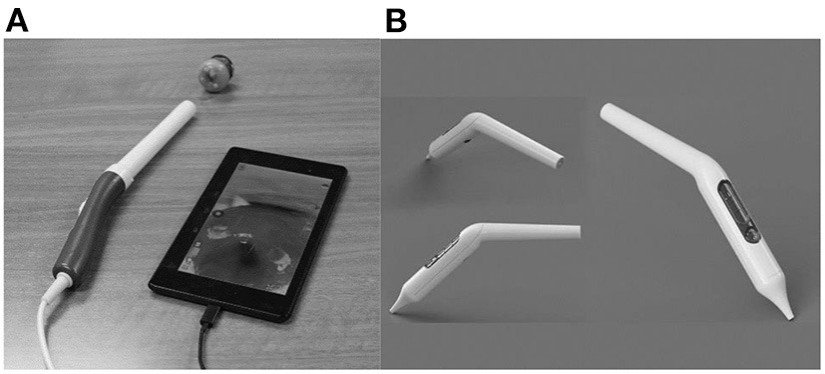
**(A)** 5-mega pixel POCkeT. **(B)** 2-mega pixel POCkeT.

The pocket colposcope can be inserted through the speculum to provide a close-up view of the cervix to take a picture. When the colposcope is close to the cervix, a set of high-quality pictures are obtained that are better than that of colposcopes on the market, and are both effective in cost and size. As seen in Figures 5A,B there are two versions of the pocket colposcope (20), one with a 5-mega pixel camera that can be used to obtain images *via* insertion through a speculum and one with a 2-mega pixel camera that is more slender and can be inserted into a tampon-like introducer called the Calla scope, to enable speculum-free visualization of the cervix.

#### TruScreen

**Figure 6:**
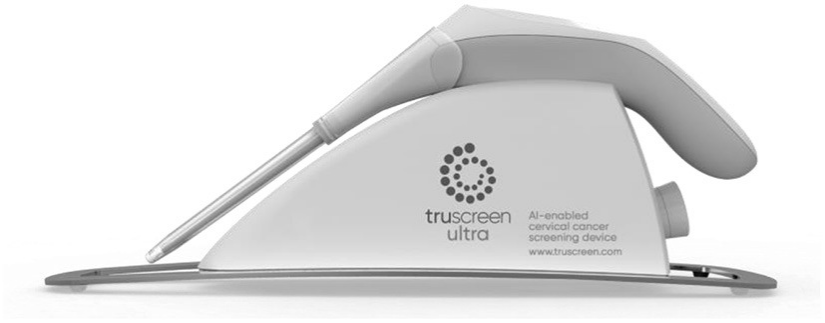
TruScreen device.

TruScreen is a unique, proprietary Opto-Electrical technology to evaluate the tissue of the cervix. Unlike cytology, TruScreen does not only examine surface epithelial cells, it produces specific frequencies of light transmitted through the cervical tissue to identify changes in the basal and stromal layers. There are four LEDs that sequentially emit light at three wavelengths, distant red, infrared and green. Electrical measurements test the cell's resistance to current to characterize the tissue. This characterization of these tissues is called electrical impedance spectroscopy (21). As seen in Figure 6, the TruScreen system consists of a disposable Single Use Sensor (SUS), a Handheld Device (HHD), and an Intelligent Cradle (IC) that work in concert to detect and classify the cancerous and precancerous changes in the cervix.

First, many areas on the cervix are gently touched using a pen-like wand wrapped in a SUS. The SUS has electrodes and a precision lensthat interact with the cervix. During this process, it transmits and receives low-level optical and electrical information from the cervical tissue.

The signals are then analyzed by an integrated AI-enabled algorithm on the TruScreen Handheld Device, which compares them to a database of 2,000 patients from various ethnic and geographic backgrounds who have different histology diagnoses. Physicians receive immediate results from this analysis, which detects the presence of abnormal (cancerous and precancerous) cells in the cervix. In contrast to traditional Pap tests, which can take weeks or even months to provide a result in some countries, each TruScreen examination produces results in 1–2 min.

#### ZedScan

**Figure 7:**
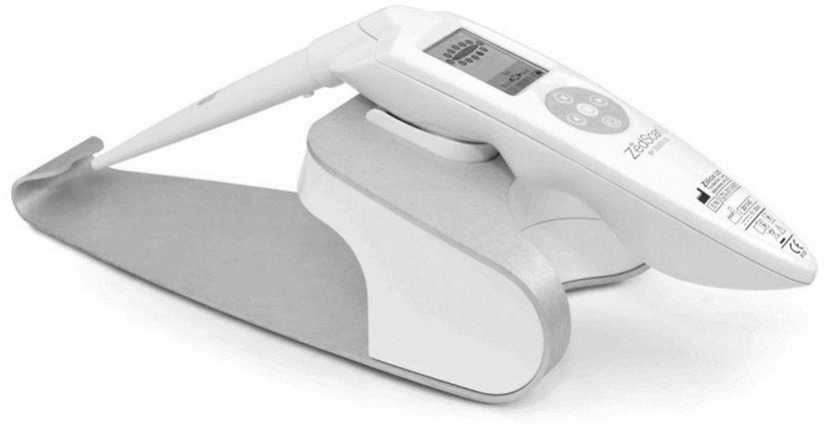
The ZedScan cervical probe.

ZedScan is a diagnostic gadget that makes use of an accessory to conventional colposcopy to offer an evaluation of the cervical epithelial tissue in real time. Electrical Impedance Spectroscopy (EIS) is a scientifically-proven technique to distinguish among normal, pre-cancerous and cancerous tissues (neoplasias) (8). This technique is likewise suitable for the prognosis of diverse cancers and pre-cancerous conditions. Figure 7 shows the ZedScan cervical probe.

ZedScan makes use of EIS technique to distinguish among normal, pre-cancerous and cancerous tissue at the cervix based on electrical properties. When used along with colposcopy, ZedScan has established extra accuracy in detecting cervical disease.

#### CervAstra

**Figure 8:**
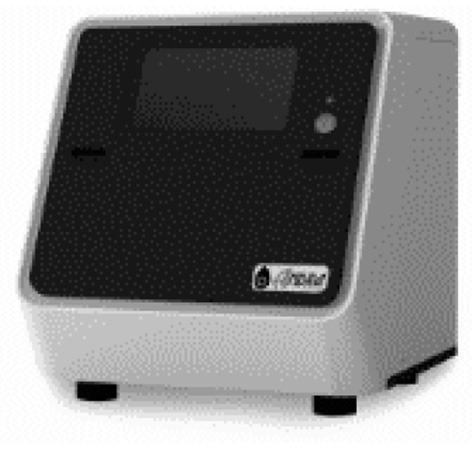
CervAstra.

There are many devices that detects and screens cervical cancer and Pap smear is one of the predominant ways. One of the greatest disadvantages of the Pap smear is that it takes a long time to receive the test results. In order to cater to this problem CervAstra was invented (22). CervAstra is a device to detect Cervical Cancer using a Computational Pathology platform. Figure 8 shows the device.

CervAstra analyzes Pap smear samples at the Point-of-Care to state normal or abnormal in few hours compared to a longer duration depending on the location of sample collection.

#### LuViva

**Figure 9:**
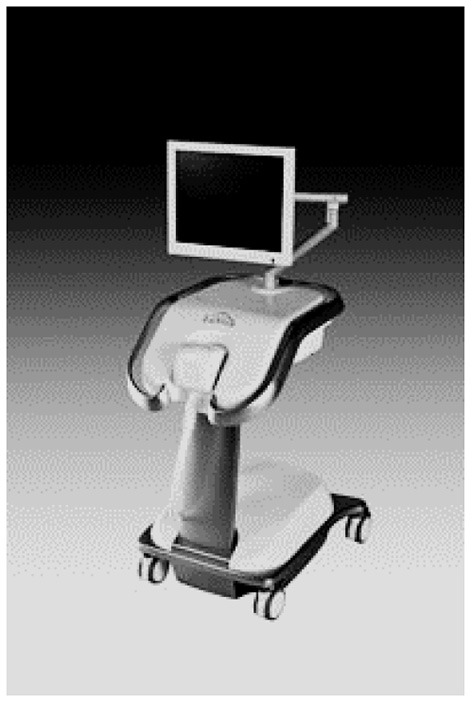
LuViva Scan device.

It is a Hyperspectral Imaging Spectroscopy (HIS) technology based non-invasive scanning device that includes a base unit and a single-patient-use disposable probe. It is useful to scan the cervix with light source to detect the cancerous and pre-cancerous cells (23). Light reflected from the cervix is analyzed through a spectrometer. Figure 9 shows the LuViva Scan device.

Based on the information from the spectrometer, an image of the cervix will be generated which distinguishes the healthy from diseased tissue. The development of this technology has yielded seventeen patents.

### Screening using AI and ML based applications

Numerous automatic and semi-automatic techniques have been developed as a result of the automatic analysis of colposcopy for the diagnosis of precancerous lesions. Neoplasia can be divided into several categories, and acetowhite zones can be classified as high- or low-risk, malignant or non-cancerous, normal or aberrant.

Many research works have been carried out to detect the cancer from Pap smear images and colposcopy images using ML and Deep Learning (DL) techniques. Many research works used Support Vector Machine (SVM) (24, 25), Adaptive Neuro Fuzzy Inference System (ANFIS) based classifier (25, 26), Bayesian classifier (27, 28) for cervical cancer detection and classification into cancerous or noncancerous. Many other ML techniques such K-Nearest Neighbor (KNN), Neural Networks, Adaboost classifier have been explored for the detection purpose.

By analyzing digitalized Papanicolaou-smear images with a primary training dataset, 15 different machine learning algorithms were built for the detection of cervical cancer. Almost all algorithms successfully identified the cancer cells. Although multilayer perceptrons are the highest among all the algorithms used in recent times multiple back propagation neural networks had a higher level of efficiency, whereas the other algorithms has a lower level of efficiency. The findings show that techniques based on AI can be utilized to develop tools for widespread cervical cancer screening (29).

Using colposcopy images, the traditional methods based on image processing and machine learning produced good results. However, these techniques require manual skill for feature extraction. The features can be automatically extracted from the data by deep learning. Apart from conventional ML techniques, existing DL architectures such as LeNet, VGG16/19, ResNet, MobileNet, Long Short-Term Memory (LSTM) and many proposed convolutional neural networks (CNN) have been used for the classification purpose.

The three most prominent Deep CNNs (ResNet-50, MobileNet, and NasNet) have been configured for training to create linearly separable image feature descriptors in which the collected deep features are used to train a KNN classifier (30). In Ref. (31), the researcher has applied DL techniques to a new dataset acquired using smartphone, hand-held device and colposcope.

A hybrid method for the classification of cervical cells using deep learning-based segmentation and an ensemble-based classifier is proposed in Ref. (32) on a publicly available dataset. The model had an average accuracy and AUC of 99.7% and 0.996 for two-class classification and 75.55% and 0.909 for four-class classification, respectively.

Gated recurrent units (GRU) is applied in Ref. (33) to build a clinical event prediction model based on recurrent neural network (RNN). The results demonstrate that RNN-2-DT has a superior predictive effect compared to traditional models that directly predict clinical events.

Although AI is an advantage of the present digital era it also has major processing problems that might not give a complete solution for cancer screening techniques. It can be used for primary level of screening through which the presence of cervical cancer can be monitored. The future awaits for more development in AI algorithms that would facilitate in secondary level of cervical cancer screening.

### Screening using mobile

From the different methodologies and studies done this far, it also required to explore the techniques based on mobile screening. With a growing technology based on mobile and smartphone, there are two major mobile based techniques in order to screen cervical cancer through smartphones, these are discussed in detail as follows.

#### Gynocular

**Figure 10:**
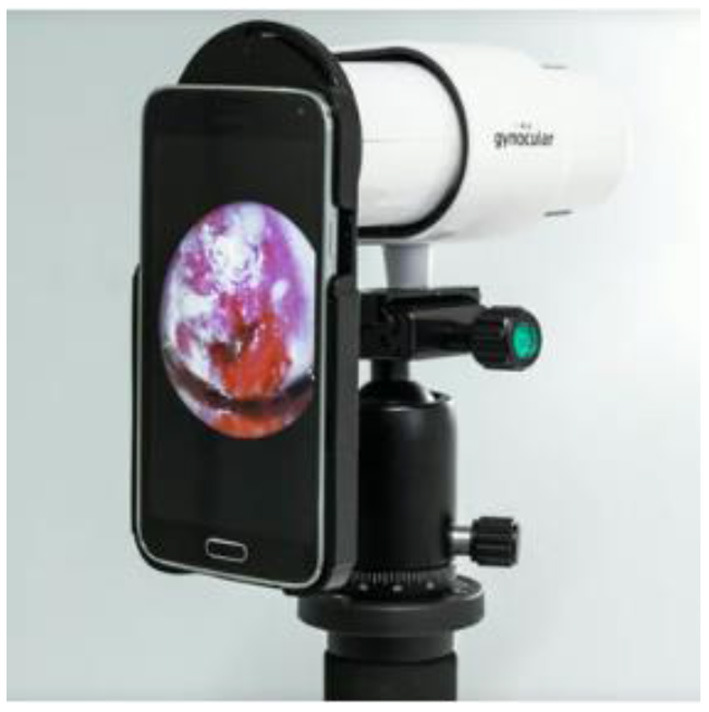
Gynocular.

The monocular colposcope called the Gynocular as shown in Figure 10 is a device that has optical capabilities when compared to basic colposcopes. This device screens the cancer using high resolution lens and is almost a smaller version of the traditional colposcope (34).

Using this device diagnostic forecasts from distant assessment were revealed to be equivalent to estimates from actual colposcopy evaluation for the diagnosis of CIN2+ lesions.

#### Mobile ODT

This device uses a method called the Enhanced Visual Assessment (EVA) Colpo, which is made up of a Mobile phone attached with magnifying lenses, a number of rechargeable batteries and LEDs for illumination. Mobile ODT has also developed a mobile app that retains patient information, preserve cervical pictures, and keeps track of biopsies along with other clinical findings (35).

**Figure 11:**
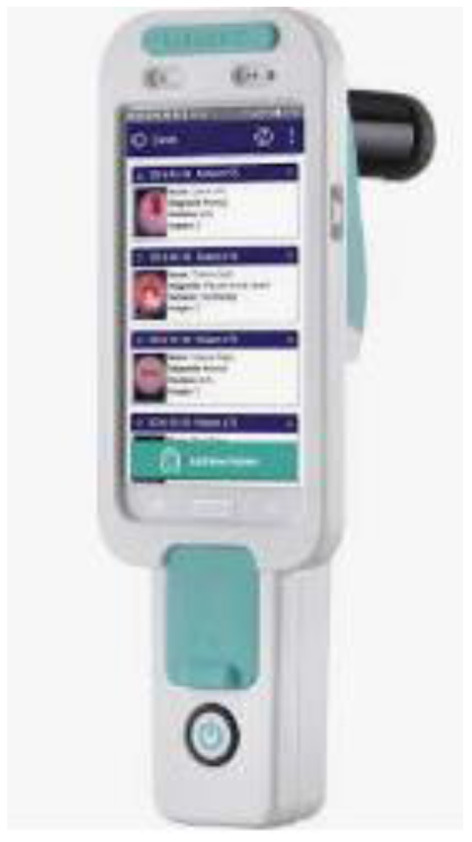
Mobile ODT.

Figure 11 shows the Mobile ODT device, this device has also proved to reduce false positive as well as false negative rates when compared to Pap tests, and the AI created in-house has been demonstrating good results.

#### Smartscopy

The ideology of smartscopy is that, instead of using an external flash and a device separately, the smartphones are used. This method is done after the application of a 3% solution of acetic acid to the cervix for 1 min, once the application is over a gynecologist inspects the cervix using Smartscopy with the activated flash mode pictures of the cervix (36). The recorded prominent areas revealed abnormal epithelium. Subsequently, the smartscopy findings, and the histological diagnosis was evaluated and was relatively successful. Although results were successful by using the iPhone 5S to inspect the uterine cervix for cervical cytology is welcoming, it might not be always always useful for screening cervical cancer.

## Conclusion

This paper intended to study various techniques that were found to be successful, simple and cost effective when it came to screening cervical cancer. In equipments like digital colposcope and LuViva which are high in cost and sensitive in hardware are difficult to be moved to rural areas. This is a disadvantage caused due to whichtraditional ways have to be followed in rural areas. Though the knowledge of how different techniques and methods have been of great use in cervical cancer screening was studied. In future, studies may have to come up methods where all of the possible screening methods are put under one roof. A cost-effective method with the application of Machine learning techniques collaborated with a mobile application would be of great use, such a device would be both cost, time efficient compared to the effectiveness of other devices. When such applications become a reality, it would be of great use in remote sectors of many developing countries.

## Author contributions

KR carried out the conceptualization of the research idea and supervised the study. GR carried out the analysis of the screening methods and verified the manuscript. SJ validated the systematic review. ST did a detailed survey on the screening methods for cervical cancer and drafted the manuscript. PE carried out the literature survey of screening techniques and methods. LM performed the study and drafted the manuscript. All authors checked and confirmed the manuscript finally.

## Funding

We are gratefully acknowledging the financial support for this work by Department of Science and Technology-Biomedical Device and Technology Development (DST-BDTD) [Grant No:TDP/BDTD/30/2021(G)].

## Conflict of interest

The authors declare that the research was conducted in the absence of any commercial or financial relationships that could be construed as a potential conflict of interest.

## Publisher's note

All claims expressed in this article are solely those of the authors and do not necessarily represent those of their affiliated organizations, or those of the publisher, the editors and the reviewers. Any product that may be evaluated in this article, or claim that may be made by its manufacturer, is not guaranteed or endorsed by the publisher.
